# Measurement of Neutrophil Gelatinase-Associated Lipocalin Concentration in Canine Cerebrospinal Fluid and Serum and Its Involvement in Neuroinflammation

**DOI:** 10.3389/fvets.2019.00315

**Published:** 2019-09-18

**Authors:** Nina Meyerhoff, Karl Rohn, Regina Carlson, Andrea Tipold

**Affiliations:** ^1^Department of Small Animal Medicine and Surgery, University of Veterinary Medicine Hannover, Hanover, Germany; ^2^Institute for Biometry, Epidemiology and Information Processing, University of Veterinary Medicine, Hanover, Germany

**Keywords:** neutrophil gelatinase-associated lipocalin, chemokine, neuroinflammation, autoimmune disease, biomarker, steroid-responsive meningitis-arteriitis, meningoencephalitis of unknown origin, canine

## Abstract

Neutrophil gelatinase-associated Lipocalin (NGAL) is a glycoprotein involved in inflammation acting as an acute phase protein and chemokine as well as a regulator of iron homeostasis. NGAL has been shown to be upregulated in experimental autoimmune encephalomyelitis (EAE) in mice. Increased NGAL concentration in cerebrospinal fluid (CSF) and expression in central nervous system (CNS) has been described in human neuroinflammatory disease such as multiple sclerosis and neuropsychiatric lupus as well as in bacterial meningitis. We aimed to investigate involvement of NGAL in spontaneous canine neuroinflammation as a potential large animal model for immune- mediated neurological disorders. A commercially available Enzyme-linked Immunosorbent Assay (ELISA) for detection of canine NGAL was validated for use in canine CSF. Concentration in CSF and serum of canine patients suffering from steroid- responsive meningitis- arteriitis (SRMA), Meningoencephalitis of unknown origin (MUO), different non- inflammatory CNS disease and control dogs were compared. Relationship between NGAL concentration in CSF and serum and inflammatory parameters in CSF and blood (IgA concentration, total nucleated cell count (TNCC), protein content) as well as association with erythrocytes in CSF, duration of illness, plasma creatinine and urinary leucocytes were evaluated. In dogs with SRMA and MUO, CSF concentration of NGAL was significantly higher than in dogs with idiopathic epilepsy, compressive myelopathy, intracranial neoplasia and SRMA in remission (*p* < 0.0001). Patients with acute SRMA had significantly higher levels of NGAL in CSF than neurologically normal controls (*p* < 0.0001). Serum NGAL concentrations were significantly higher in dogs with SRMA than in patients with myelopathy and intracranial neoplasia (*p* < 0.0001). NGAL levels in CSF were strongly positively associated with IgA concentration (rSpear= 0.60116, *p* < 0.0001), TNCC (rSpear= 0.65746, *p* < 0.0001) and protein content (rSpear= 0.73353, *p* < 0.0001) in CSF. It can be measured in CSF of healthy and diseased dogs. Higher concentrations in canine patients with SRMA as well as positive association with TNCC in CSF suggest an involvement in pro-inflammatory pathways and chemotaxis in SRMA. High serum levels of NGAL in serum of SRMA patients in different stages of disease might reflect the systemic character of the disease.

## Introduction

Neutrophil gelatinase-associated Lipocalin (NGAL) or Lipocalin-2 (LCN2), is a versatile glycoprotein acting as an acute-phase protein as well as part of the innate immune system mainly by its function as a siderophore-binding protein (1). It is produced and stored in neutrophils (2), macrophages (3), but also in astrocytes (4, 5) and the choroid plexus (6) and many other tissues (7, 8). In the central nervous system (CNS), neuroprotective (9, 10), as well as detrimental impact during neuroinflammatory processes has been described (11–13). NGAL induces astrocytosis (13, 14), and enhances immune cell migration (15–17) especially via recruitment of neutrophils (18, 19).

Increased NGAL concentration was found in cerebrospinal fluid (CSF) of human patients with bacterial meningitis (20, 21) but also in patients with autoimmune CNS disease such as multiple sclerosis (22, 23) and neuropsychiatric lupus (24). It was also detected in brain tissue in experimental animal studies after application of lipopolysaccharides (25) and has been shown to be upregulated in mice with experimental autoimmune encephalomyelitis (EAE) which serves as a model for human multiple sclerosis (5).

Until now, data regarding concentration of NGAL in CSF of healthy dogs and canine patients with inflammatory and non-inflammatory CNS disease is lacking. Further investigation of spontaneous inflammation of the central nervous system in dogs (26–28) could be useful for closing the gap between experimental animal models and investigation of human spontaneous autoimmune neuroinflammation.

This study aimed to test the hypothesis that NGAL concentrations in CSF would be higher in steroid-responsive meningitis-arteriitis (SMRA) and MUO (Meningoencephalitis of unknown origin) than in non-inflammatory (idiopathic epilepsy, intracranial neoplasia, intervertebral disc disease) CNS disease and neurological healthy dogs. We also hypothesized that there would be a correlation with the total nucleated cell count (TNCC) in CSF, possibly due to chemotaxis.

The overall goal of the study is improvement of differentiation of CNS diseases and amelioration of the understanding of the etiopathology of suspected autoimmune canine CNS disease like SRMA and MUO by detection of a new biomarker in canine CSF. Better understanding of chemokine signaling pathways and modulation of neuroinflammation could help to develop more specific treatment strategies for aforementioned pathologies (5, 13, 29, 30).

## Materials and Methods

### Serum and Cerebrospinal Fluid Samples

Paired serum and CSF samples were collected for routine diagnostic workup between December 2011 and May 2018 at the Department for Small Animal Medicine and Surgery of the University of Veterinary Medicine, Hannover, Germany, from client–owned dogs presented to the neurology group and healthy, university-owned beagles (animal experiment number 33.9-42502-05-14A453). The specimens were aliquoted and stored at −20°C prior to measurement of NGAL concentrations. The study was conducted according to the University's ethical guidelines.

### Animals

The dogs underwent general and neurological examination, complete blood cell count (CBC), blood chemistry, CSF analysis including IgA measurement and individual combination of further specific testing (urine analysis, orthopedic examination, radiographs, testing for infectious agents in CSF and/or blood, advanced diagnostic imaging such as magnetic resonance imaging or computed tomography, abdominal or heart ultrasound, electrodiagnostics, surgery, histopathology) to obtain a definitive or highly likely diagnosis (**Table 2**).

Dogs were retrospectively assigned to different groups regarding diagnosis: SRMA acute (untreated), SRMA recurrence, SRMA in remission (under therapy with glucocorticosteroids), MUO (including confirmed and presumed cases, see Supplementary Table 4), intracranial neoplasia, compressive myelopathy [intervertebral disc herniation (IVDH) or spondylomyelopathy], idiopathic epilepsy (IE), others (behavioral abnormalities, idiopathic vestibular syndrome, bacterial meningoencephalitis). The diagnoses were defined according to published data (**Table 2**).

Healthy dogs and dogs with final diagnosis of orthopedic disease with a normal general and neurological examination and laboratory values in the reference range served as control group (“healthy/orthopedic”).

Tissue samples were obtained following routine necropsy after spontaneous death or euthanasia on owner's request due to poor prognosis and were conducted in accordance with the German Animal Welfare Act within the law of animal welfare, Germany, and following the ethical guidelines of the University of Veterinary Medicine Hannover. No dogs were euthanized for this particular study; sample aliquots were obtained for clinical diagnostics or previously attained during other studies (animal experiment number 33.9-42502-05-14A453).

### Data Collection

Information regarding duration of clinical signs, comorbidities, signalment, and course of disease as well as blood and urine parameters and if available biopsy or necropsy results were retrospectively evaluated using the digital clinical data software (easyVET, VetZ GmbH, Isernhagen, Germany). If possible, follow up was conducted via control appointments or phone calls to owners or local practitioners (see Supplementary Table 2 for signalement, urine, and renal parameters).

### Quantification of Neutrophil Gelatinase-Associated Lipocalin

A commercially available Sandwich Enzyme-linked immunosorbent Assay (ELISA) established for canine NGAL in urine, plasma, serum, tissue extracts, or culture media (36–40) was used following manufacturer's instructions (Dog NGAL ELISA Kit 043, Bioporto, Hellerup, Denmark). It was validated for measurement of NGAL- concentration in pooled, aliquoted and frozen canine CSF by determination of intraassay and interassay reproducibility and analysis of recovery rate using CSF samples spiked with calibrator fluid. Intraassay reproducibility was calculated by measuring pooled CSF in seven replicates (each as duplet) and calculation of the coefficient of variance (CV). Interassay reproducibility was tested calculating CV for pooled and aliquoted CSF measured in duplicates on five different dates.

Concentration of NGAL in paired serum and CSF samples was afterwards measured following manufacturer's instructions. The mean minimum detectable value given by the manufacturer was 0.56 pg/mL. If the value exceeded the highest value of the standard curve (400 pg/mL), the sample was diluted and measured again. All samples were analyzed in duplicates and mean values were calculated.

### Quantification of Immunoglobulin A

IgA in aliquoted serum and CSF samples was measured prior to this study as part of CSF analysis in-house using an ELISA as previously described (41).

### Data Analysis

Data was analyzed using the statistical software SAS Enterprise Guide 9.3 (SAS Institute Inc., Cary, NC, USA). The Kolmogorov–Smirnov-Test was used to analyze the data for normal distribution. This was repeated after identification and exclusion of outliers. Because data did not conform to Gaussian distribution, non-parametric tests consisting of Kruskal–Wallis test, Dunn's-*post hoc* test and Wilcoxon two-sample test were performed. Wilcoxon two-sample test was used to compare all groups pairwise (see Supplementary Table 1). Values of *p* < 0.0001 were considered significant when comparing medians of the means. Spearman's rank correlation coefficients were calculated to analyze associations between NGAL concentration in serum and CSF, IgA concentration, and NGAL concentration in serum and CSF, NGAL concentration and nucleated cell count in CSF, NGAL concentration in CSF and erythrocyte count in CSF, NGAL concentration and duration of illness in inflammatory disease, NGAL concentration in serum and creatinine concentration in blood plasma, and NGAL concentration in serum and presence of leucocytes in urine detected by Combur stick (Roche Deutschland Holding GmbH, Mannheim, Germany). As stability of NGAL in frozen canine samples has not been reported, linear regression analysis by groups was conducted to identify possible influence of sampling year and storage period on NGAL concentration in canine CSF and serum. Scatter graphs from the obtained data were created using GraphPad software (GraphPad Prism™ ®, version 5, La Jolla, CA, USA).

## Results

### Validation of ELISA for Use of NGAL Measurement in Cerebrospinal Fluid

Recovery rate of the four CSF samples spiked with calibrator fluid is shown in Table 1. For intraassay reproducibility, the coefficient of variance (CV = 3.9%, median NGAL concentration 387.85 pg/ml) was calculated. Interassay reproducibility was tested calculating CV for pooled CSF (CV = 6.2%, median NGAL concentration 648.875 pg/ml).

**Table 1:** Recovery rate of four CSF dilutions spiked with calibrator fluid. Recovery was calculated as (“Measured”/“Calculated”) × 100%.

**Sample**	**Measured (pg/mL)**	**Calculated (pg/mL)**	**Recovery (%)**
CSF1	32.395	28.2	114.87
CSF2	68.547	68.2	100.5
CSF3	117.019	118.2	99.0
CSF4	233.076	218.2	106.82
		Mean recovery	105.3

### Canine Cerebrospinal Fluid and Serum Samples

In total, 163 CSF and 157 paired serum samples of 140 individual patients were included (Table 2). Twenty-five of the patients in the acute SRMA group also had samples included in “SRMA therapy” and three had samples included in “SRMA recurrence.” One SRMA patient only had samples analyzed as a part of the SRMA recurrence group, but SRMA acute and SRMA control samples were not available. All other patients had only samples from one time point included.

**Table 2:** Distribution of number of cases and samples in different disease and control groups.

**Disease categories**	**Number of samples (CSF/Serum)**	**Number of dogs (individuals)**
**SRMA acute (untreated)**		
Dogs with cervical hyperesthesia, fever, neutrophilic leukocytosis and pleocytosis, elevated IgA levels in CSF and serum (38,39,36)	35/34	36
**SRMA therapy**		
Dogs previously diagnosed with SRMA under long-term treatment with glucocorticoids and remission of clinical signs	25/24	25
**SRMA recurrence**		
Dogs previously diagnosed with SRMA showing clinical relapse after weaning of GC to a non-immunosuppressive dosage or after discontinued therapy	4/4	4
**Meningoencephalitis of unknown origin**	17/15	
Dogs with clinical, CSF, MRI, and/or pathological findings consistent with meningoencephalitis of unknown origin (31)	Histopathology *n* = 4/17 Positively tested pug dog encephalitis risk gene *n* = 1/17 Presumed *n* = 13/17	17
**Compressive myelopathy** (IVDH or malformation)		
Dogs with clinical signs, MRI/CT, CSF findings consistent with compressive myelopathy due to IVDH or anomalies (32, 33)	28/31	31
**Idiopathic epilepsy**		
Dogs with clinical signs, MRI, and CSF findings consistent with IE Tier II confidence level (34)	24/22	24
**CNS neoplasia**	21/19	
Dogs with clinical, CSF, MRI, and/or pathological findings consistent with primary or secondary CNS neoplasia (35)	Histopathology *n* = 8/21 4/8 Meningioma 1/8 Glioblastoma multiforme 1/8 Malignant blastoma 1/8 Metastatic prostatic carcinoma 1/8 Neuroendocrine pituitary gland tumor	21
**Control** (healthy + orthopedic)	7/5	7
**Others** (bacterial meningoencephalitis, idiopathic vestibular syndrome, behavioral abnormalities)	2/3	3
Total	163/157	168 single examinations (140 individual patients)

*SRMA, steroid-responsive meningitis-arteriitis; CSF, cerebrospinal fluid; MRI, magnetic resonance imaging; IVDH, intervertebral disc herniation; IE, idiopathic epilepsy; CNS, central nervous system, GC, glucocorticosteroids*.

### Concentration of NGAL in Canine CSF and Serum

**Figure 1:**
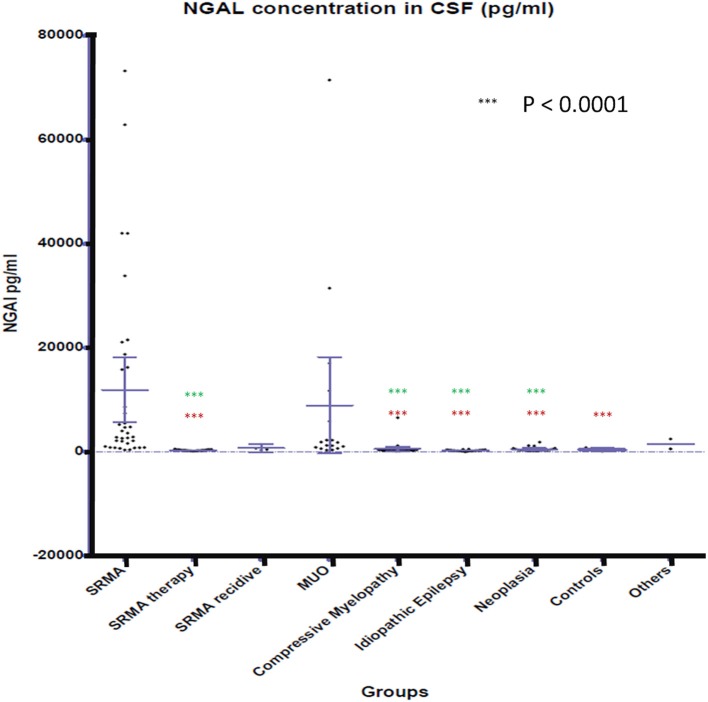
NGAL concentrations in CSF of the different groups. Boxes contain values from the first to the third quartile; lines inside the boxes indicate median values; end of vertical lines show minimum and maximum values; dots represent outliers. Red asterisks (***) represent groups differing significantly (*p* < 0.0001) from acute SRMA group. Green asterisks (***) represent groups differing significantly from MUO group. NGAL, neutrophil gelatinase-associated lipocalin; SRMA, steroid-responsive meningitis-arteriitis; CSF, cerebrospinal fluid; MUO, meningoencephalitis of unknown origin; IE, idiopathic epilepsy; “SRMA therapy” consisted of dogs under treatment without clinical signs, “SRMA recidive” consisted of dogs with relapse of clinical signs, “Myelopathy” group consisted of dogs with intervertebral disc herniation or compressive spondylomyelopathy, “Neoplasia” included dogs with primary and secondary brain tumors, “Controls” included patients without neurological disorders (healthy and orthopedic), “Others” included various neurological diseases (bacterial meningoencephalitis, idiopathic vestibular syndrome, behavioral abnormalities).

**Figure 2:**
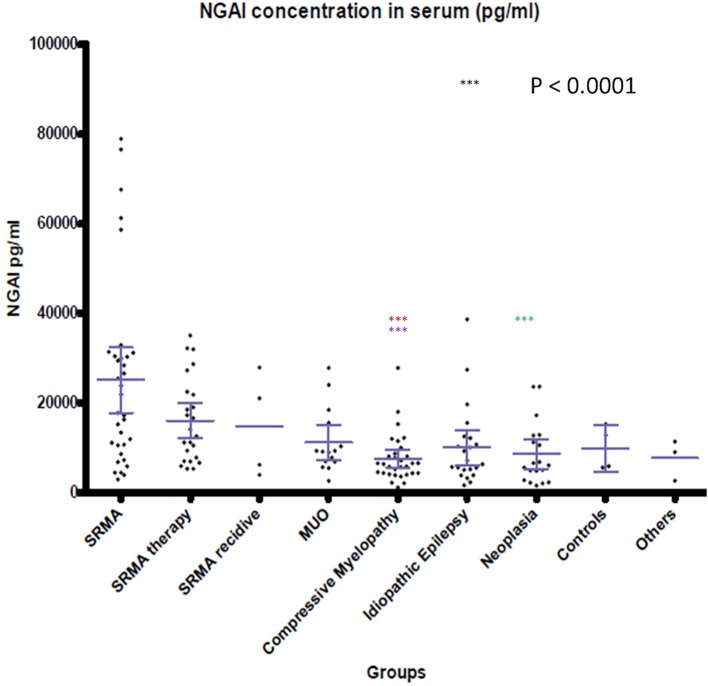
NGAL concentrations in serum of the different groups. Boxes contain values from the first to the third quartile; lines inside the boxes indicate median values; end of vertical lines show minimum and maximum values; dots represent outliers. Red asterisks (***) represent groups differing significantly (*p* < 0.0001) from acute SRMA group. Lilac arterisks (***) represent groups differing significantly from SRMA therapy group. Green arterisks (***) indicate groups differing significantly from SRMA acute group only in Dunn's *post hoc* test. NGAL, neutrophil gelatinase-associated lipocalin; SRMA, steroid-responsive meningitis-arteriitis; CSF, cerebrospinal fluid; MUO, meningoencephalitis of unknown origin; IE, idiopathic epilepsy; “SRMA therapy” consisted of dogs under treatment without clinical signs, “SRMA recidive” consisted of dogs with relapse of clinical signs, “Myelopathy” group consisted of dogs with intervertebral disc herniation or compressive spondylomyelopathy, “Neoplasia” included dogs with primary and secondary brain tumors, “Controls” included patients without neurological disorders (healthy and orthopedic), “Others” included various neurological diseases (bacterial meningoencephalitis, idiopathic vestibular syndrome, behavioral abnormalities).

NGAL was detected in all available CSF and serum samples of patients of the different groups. NGAL concentrations in CSF and Serum of the disease groups and controls are shown in Figures 1, 2. Table 3 reveals median concentrations of NGAL (pg/ml) in canine CSF and serum as well as ranges. The highest concentrations of NGAL in CSF were found in dogs with acute SRMA and MUO. Canine NGAL serum concentration was overall higher than in CSF with highest concentration in dogs with acute SRMA, SRMA in remission under therapy and SRMA recurrences.

**Table 3:** Median concentrations and ranges of NGAL (pg/ml) in canine CSF and serum in different groups.

**Disease categories**	**Number of samples (CSF/Serum)**	**Median NGAL pg/ml CSF**	**Median NGAL pg/ml Serum**	**Range NGAL pg/ml CSF**	**Range NGAL pg/ml Serum**
**SRMA acute** (untreated) *UTI cases n = 3/36*	35/34 *3/3*	3603.36 *18742.019*	19834.04 *29946.5*	364.59–73241.29 *364.586–21518.38*	2980.93–78926.07 *10714.84–76587.575*
**SRMA therapy**	25/24	298.19	13361.02	140.62–536.24	5310.48–35063.71
**SRMA recurrence**	4/4	552.19	13648.30	322.25–1432.94	3982.01–27906.87
**Meningoencephalitis of unknown origin**	17/15	1786.24	9171.12	315.18–71463.69	2647.38–27839.06
**Compressive myelopathy** *UTI cases n = 3/31*	28/31 *1/3*	268.45 n = 1; 568.7*	6382.71 *6564.45*	128.19–6551.73 *not applicable*	1159.57–27837.87 *2234.16–10023.065*
**Idiopathic epilepsy**	24/22	246.85	6748.95	6.19–528.68	1653.58–38652.95
**CNS neoplasia** *UTI cases n = 5/21*	21/19 *5/5*	355.92 *414.261*	6115.54 *6596.795*	152.22–1848.98 *275.671–689.429*	1582.53–23655.50 *2804.125–23655.5*
**Control** (healthy + orthopedic)	7/5	428.33	9867.42	95.72–801.042	5605.82–15277.60
**Others** (bacterial meningoencephalitis, idiopathic vestibular syndrome, behavioral abnormalities)	2/3	1524.91	9028.75	567.54–2482.29	2662.11–11431.13
Total	163/157				

SRMA, steroid-responsive meningitis-arteriitis; NGAL, neutrophil gelatinase-associated lipocalin; CSF, cerebrospinal fluid; MRI, magnetic resonance imaging; IE, idiopathic epilepsy; CNS, central nervous system; *Sample only available in one patient, therefore no median calculated. Italic values indicates UTI cases (UTI, urinary tract infection).

Kruskal–Wallis-Test indicated a significant difference in CSF NGAL levels [χ^2^(8) = 95.9672; *p* < 0.0.0001] between the different groups. Wilcoxon rank sum test results are shown in Supplementary Table 1. NGAL concentrations in CSF differed significantly (*p* < 0.0001) between acute SRMA patients and the group with dogs displaying SRMA in remission under corticosteroid therapy, the group with idiopathic epilepsy, intracranial neoplasia, compressive myelopathy and the control group. NGAL concentrations in CSF were significantly higher in dogs with MUO than in SRMA patients in remission under therapy, idiopathic epilepsy patients, the compressive myelopathy group as well as than in dogs suffering from intracranial neoplasia (*p* < 0.0001; Supplementary Table 1).

Kruskal- Wallis test identified significant differences for NGAL serum concentration when comparing the nine groups [χ^2^(8) = 36.4667; *p* < 0.0001]. Using Wilcoxon-ranked sum test, in dogs with acute SRMA and SRMA in remission, serum levels of NGAL were significantly higher than in serum of dogs with compressive myelopathy (*p* < 0.0001; Supplementary Table 1). Additionally, Kruskal–Wallis-Dunn's test identified a difference between NGAL serum levels in dogs with acute SRMA and intracranial neoplasia [difference = 93.42, SE = 12.51, *q* = 7.47, *q*_(0.05)_ = 3.197].

### Correlation Analysis

As shown in Table 4, NGAL levels in CSF and serum were weakly positively associated. NGAL concentration in CSF, but not in serum was strongly positively associated with IgA concentration in CSF and serum, respectively. There was a strong, positive association of NGAL concentration in CSF with total nucleated cell count (lc/3ul) in CSF and a strong, positive association with protein content in CSF. Erythrocyte count in CSF was weakly positively associated with NGAL concentration in canine CSF (see Supplementary Table 3 for an overview of amount of red blood cells, leucocytes, and NGAL in CSF).

**Table 4:** Association of NGAL concentration in CSF and serum with other parameters.

**Parameters**	***r_***s***_***	***p***
NGAL concentration in CSF and NGAL concentration in serum	0.29	0.0004
NGAL concentration in CSF and IgA concentration in CSF	**0.60**	**<0.0001**
NGAL concentration in CSF and protein content in CSF	**0.73**	**<0.0001**
NGAL concentration in serum and IgA concentration in serum	−0.12	0.1358
NGAL concentration in CSF and total nucleated cell count in CSF	**0.66**	**<0.0001**
NGAL concentration in CSF and erythrocyte count in CSF	0.14	0.0819
NGAL concentration in CSF and duration of illness in SRMA and MUO patients	−0.18	0.1857
NGAL concentration in serum and duration of illness in SRMA and MUO patients	−0.32	0.0186
NGAL concentration in serum and creatinine in blood plasma	0.1	0.2816
NGAL concentration in serum and leucocytes in urine	0.15	0.2748

*NGAL, Neutrophil Gelatinase-Associated Lipocalin; SRMA, Steroid-responsive meningitis-arteriitis; IgA, immunoglobulin A; CSF, cerebrospinal fluid; MUO, meningoencephalitis of unknown origin. Bold values indicates statistically significant*.

There was a weak, negative association between duration of illness in inflammatory disease and level of NGAL in CSF. Duration of illness in inflammatory disease (SRMA, MUO) showed a moderate negative association with NGAL concentration in canine serum.

A weak, positive association was detected between NGAL serum levels and concentration of creatinine in blood plasma and leucocytes in urine.

### Linear Regression Analysis to Identify Influence of Sample Year on NGAL Concentration

Linear regression analysis by group did not identify significant linear regression coefficients of NGAL in CSF or serum through year of sampling (*p* > 0.05).

## Discussion

In the current study, a commercially available NGAL ELISA was validated for use in canine CSF and, for the first time, concentration of NGAL in CSF and serum in dogs with different inflammatory and non-inflammatory disease was described.

NGAL in canine CSF was shown to be elevated in dogs with inflammatory disease (SRMA and MUO) in comparison to non-inflammatory CNS disease categories (compressive myelopathy, neoplasia, IE). A positive association between NGAL concentration in CSF and TNCC in CSF was found, which could indicate enhanced chemotaxis as described in experimental studies (15–17) and increased permeability of the blood brain barrier, for example via disruption of tight junctions as described in rodent models with artificial stroke (42, 43). When assessing the different subgroups against each other, CSF levels of NGAL in MUO (confirmed and presumed cases) and acute SRMA were significantly higher than in dogs with idiopathic epilepsy, compressive myelopathy, intracranial neoplasia, and SRMA in remission. CSF levels in acute SRMA, but not SRMA recurrence and MUO were significantly higher than in control dogs without neurological disease. The group of dogs with SRMA recurrence was very small (*n* = 4) due to sample availability and rare occurrence of relapses in our SRMA population. Therefore, explanatory power is questionable for this group. MUO on the other hand represents a very heterogenous group because different entities are covered by this umbrella term (26, 28), as long as no histopathological diagnosis is available. In our study, confirmed and presumed MUO cases were included (see also Supplementary Table 4). For further studies, inclusion of histopathologically confirmed MUO cases only would be desirable.

The strong, positive association of NGAL levels in CSF and protein content in CSF could be caused by aforementioned blood brain barrier disruption and influx of proteins from the periphery or, on the other hand, be caused by increased intrathecal immunoglobulin synthesis as well as NGAL synthesis in neuroinflammation. The strong positive association of NGAL and IgA in CSF most probably reflects the local synthesis of immunoglobulins in the CNS (41) and of NGAL. Intrathecal production of NGAL in endothelial and glial cells as well as in the choroid plexus is suspected, as it was shown previously in rodent models and human samples (5, 6, 11, 19, 22, 23). Positive association of CSF NGAL and IgA levels could indicate overlapping signaling pathways. Chemokines and proteins which have been described to play an important role in SRMA are Il-6 (44), IL-8 (45), IL-17 (46), MMP2 and MMP9 (47). Those have also been described in cell culture and experimental murine or porcine studies to be induced by NGAL or to increase NGAL expression themselves (48–54). In a glial cell model and murine EAE, NGAL treated cells expressed higher levels of MMP9 and upregulated MMP9 was suspected to increase blood brain barrier damage (12). Concentration of NGAL in canine CSF and serum were only weakly positively associated, additionally supporting the theory of intrathecal NGAL synthesis in dogs.

NGAL in CSF of clinically normal dogs with SRMA in remission under corticosteroid therapy, which were presented for CSF examination several weeks after initiation of therapy, did show significant decrease of levels compared to acute, untreated SRMA. Interestingly, in those dogs, serum NGAL levels remained high in the course of the disease as it has also been described for IgA levels in dogs (55, 56). This finding could be worth further investigation in a prospective study with SRMA cases only and synchronized sampling intervals, as it might help in diagnosis of atypical or protracted SRMA cases. Our findings contradict an experimental study with pigs receiving hydrocortisone prior to iatrogenic induced endotoxemia in which hydrocortisone counteracted NGAL plasma increase (57). This might be due to different time points of measurement as plasma NGAL concentration in the experimental study was only sampled up to 6 h after hydrocortisone application whereas in our study, sampling took place before steroid treatment in the acute SRMA group and weeks or months after initiation of corticosteroid therapy in the dogs with SRMA in remission under therapy.

NGAL levels in serum differed significantly between acute SRMA cases and SRMA patients in remission when compared to compressive myelopathy patients, but this was not found in MUO patients. In contrast to MUO, SRMA is considered a systemic disease and is associated with neutrophilic pleocytosis in CSF and peripheral blood (26, 27, 41). Studies on murine and human cell cultures and experimental studies with lcn2 knockout mice revealed an especially high impact of NGAL on neutrophil function (16, 17). MUO is not a homogenous pathology as it includes different diseases like granulomatous meningoencephalitis or necrotizing encephalitis (26, 28), which is typically associated with mononuclear pleocytosis in CSF but not necessarily pleocytosis in peripheral blood (31). This might explain why median ranked values of serum NGAL levels in MUO patients did not differ significantly in statistical analysis compared to the controls and non-inflammatory disease groups. Dunn's Kruskal–Wallis *post-hoc* test also identified a significant difference between serum samples of acute SRMA and intracranial neoplasia. This could as well-represent the systemic nature of SRMA in contrast to intracranial neoplasia as only one of the included patients suffered from metastatic neoplastic disease (prostatic carcinoma). However, this result should be considered with caution, as there was detectable overlap of concentrations in those two groups as can be seen in Figure 2. Apart from that, no statistically significant differences between the remaining serum groups were detected. This could reflect heterogeneously composed groups not only in MUO patients but also in intracranial neoplasia and the compressive myelopathy group, where different entities, combined with possible underlying diseases as well as acute and more chronic myelopathies were included, possibly leading to different grades of disease severity at timepoint of sampling. Medians and ranges of serum levels were overall comparable to published data regarding serum NGAL levels in dogs (36, 58–60) but our results raise the suspicion, as postulated before (58, 61), that higher levels of NGAL in serum alone might not be specific for renal disease due to its complex role in a variety of other peripheral inflammatory and neoplastic pathologies. In the future, further studies comparing NGAL concentration in serum in systemic inflammatory, renal and other diseases could be useful. As NGAL is also acting as a regulator of iron metabolism (1) and might be increased in case of hemorrhage, which is possible in SRMA (62) because of necrotizing vasculitis, we investigated if there is an association of erythrocyte content and NGAL levels in CSF (see also Supplementary Table 3). For those parameters, only a weak, positive association with questionable relevance was detected, so NGAL levels might not always be influenced by hemorrhage depending for example on chronicity of disease or the erythrocytes detected in our CSF samples might have been present due to iatrogenic contamination. Further studies should include a group of patients with intracerebral hemorrhage or vascular incidents to investigate NGAL CSF levels in CSF and serum in those patients.

In humans and dogs, serum and urinary NGAL have been investigated as an early marker of renal dysfunction (36, 59, 63) and urinary NGAL was shown to be increased in dogs with urinary tract infection (37). Therefore, we also included data regarding renal parameters and urine analysis (see Supplementary Table 2) but found only a weak, positive association between NGAL concentration in serum and plasma creatinine levels or leucocytes in urine. Nevertheless, statistical evaluation could be biased as only a small number of dogs included in the study were diagnosed with renal disease (*n* = 2, diagnosed by histopathology) and none of the patients showed increased plasma creatinine at time of sampling for CSF and serum. Sixteen dogs had clinical signs of urinary tract infection (UTI) at time of sampling (five dogs with intracranial neoplasia, three dogs with acute SRMA and compressive myelopathy each, two dogs diagnosed with IE and one each in the groups of SRMA in remission, SRMA recurrence and MUO, see Supplementary Table 2).

Outliers, which showed markedly increased NGAL concentration in CSF were identified. In the acute SRMA group, one of the highest measured NGAL concentration belonged to a patient who later relapsed after reduction of prednisolone dosage, but the other three outliers either had a good outcome (follow up period 7 and 17 months, respectively) or were lost to follow up (*n* = 1). In the MUO group, the patient with highest NGAL concentration was euthanized directly after diagnosis on owner's request and an unusual, marked sterile pyogenic meningoencephalitis was found in necropsy. Nevertheless, the other two outliers had a good or satisfying outcome. One patient with very high NGAL CSF levels in the group with compressive myelopathy initially had a good outcome but developed another intervertebral disc herniation at another location 4 months later and the other outlier was a patient who had undergone hemilaminectomy 1 year prior. One of the patients with very high NGAL levels in CSF in the neoplasia group had a suspected choroid plexus tumor, an interesting fact, as the choroid plexus has been described as one of the main intrathecal areas of NGAL synthesis (6). No histopathological confirmation of tumor type was available in this dog. In one other patient with very high NGAL CSF levels, histopathology after euthanasia on owner's request identified meningioma with secondary surrounding encephalitis in the brainstem. The third outlier showed central vestibular signs and had an extraaxial cerebellar mass with suspicion of meningioma, but necropsy was not provided.

In serum, one very high NGAL concentration in the acute SRMA group belonged to a patient who relapsed under reduced prednisolone dosage, but the other two patients had a good outcome (in both patients follow up period 2 months). No remarkable features were detectable in the other outliers regarding NGAL in serum in the myelopathy and idiopathic epilepsy group, who had either a good outcome or were lost to follow up.

A correlation of high serum NGAL concentration with negative outcome in patients with sepsis as well as traumatic brain injury has been described (64, 65). Therefore, in the future we aim to investigate possible correlation of NGAL concentration in serum and CSF of dogs with diverse CNS disease and their clinical outcome. Moreover, as NGAL levels in CSF differed significantly between MUO and intracranial neoplasia, it might be a promising biomarker for differentiating categories of mass lesions in canines in addition to MRI, if biopsies are declined due to risks of the procedure (66). A limitation of the presented study is the retrospective nature and that necropsy or biopsy results were not provided for all cases either due to ethical concerns of the owners or good outcome mostly in SRMA cases. Interestingly, we found detectable NGAL concentration in CSF not only in glioma as described in human tumor tissue (67), but also in confirmed meningioma cases and a metastatic brain tumor, which might indicate secondary neuroinflammation in dogs with brain tumors.

Treatment of SRMA and MUO consists of long- term immunosuppressant monotherapy with glucocorticosteroids or with additional immunomodulatory or chemotherapeutic drugs. Treatment so far is unspecific and bears risk of adverse effects as well as insufficient response (28, 61). Therefore, adapted therapy for immune-mediated neuroinflammatory diseases targeting specific signaling pathways or via modulation of gene expression is urgently needed. Different drugs were shown to decrease NGAL levels in animal models including usage of nanotherapeutics for down regulation of NGAL synthesis (29), application of the iron chelator deferoxamine in a traumatic brain injury model in rats (30) and modulation of NGAL in EAE-mice treated with natalizumab which is currently used in human multiple-sclerosis (5). The current study might provide the foundation for future treatment studies of canine neuroinflammation.

In conclusion, NGAL can be detected in canine CSF using commercially available ELISA. It is increased in CSF of dogs with SRMA compared to controls and non-inflammatory disorders and also is higher in dogs with MUO compared to dogs with idiopathic epilepsy, compressive myelopathy and intracranial neoplasia. Concentration of NGAL in CSF of dogs with SRMA in remission under therapy with corticosteroids is significantly lower than in acute SRMA- and MUO- patients. NGAL levels in canine serum were significantly higher in patients with SRMA compared to those suffering from myelopathy reflecting unspecific systemic disease, but not necessarily indicating renal failure as proposed before. NGAL is a promising biomarker in canine CSF and serum, potentially adding another piece of the puzzle to the etiopathology of autoimmune neuroinflammation.

## Data Availability

The datasets generated for this study are available on request to the corresponding author.

## Ethics Statement

Ethical review and approval was not required for the animal study because it is a retrospective study using data and samples acquired previously. Tissue samples were obtained following routine necropsy after spontaneous death or euthanasia on owner's request due to poor prognosis and were conducted in accordance with the German Animal Welfare Act within the law of animal welfare, Germany, and following the ethical guidelines of the University of Veterinary Medicine Hannover. No dogs were euthanized for this particular study; sample aliquots were obtained for clinical diagnostics or previously attained during other studies (animal experiment number 33.9-42502-05-14A453). Written informed consent was obtained from the owners for the participation of their animals in this study.

## Author Contributions

AT conceived, designed, and supervised the study. NM assisted with study design, collected and analyzed data, performed the experiments, and wrote the first draft of the manuscript. KR gave substantial contribution to the statistical analysis of data. RC conceived in part the study and gave substantial contributions to acquisition, analysis and interpretation of the data. All authors contributed to the critical revision of the manuscript for important intellectual content and have read and approved the final version.

### Conflict of Interest Statement

The authors declare that the research was conducted in the absence of any commercial or financial relationships that could be construed as a potential conflict of interest.

## Supplementary Material

Supplementary Table 1 Wilcoxon rank sum test results.

Supplementary Table 2 Summary of the signalement, creatinine concentration, leucocytes in urine, urinary tract disease, duration of illnes prior to sample date in patients with inflammatory disease.

Supplementary Table 3 Overview of NGAl level, totan nucleated cell count, and erythrocyte count in CSF.

Supplementary Table 4 Overview of MUO cases (histopathology if available, follow- up period, outcome).

## Abbreviations

CBC: complete blood count
CSF: cerebrospinal fluid
CNS: central nervous system
CV: coefficient of variance
EAE: experimental autoimmune encephalomyelitis
ELISA: enzyme-linked immunosorbent assay
Dept.: department
IE: idiopathic epilepsy
IG: immunoglobulin
IL: interleukin
IVDH: intervertebral disc herniation
lc: leucocyte
LCN2: lipocalin 2
MMP: matrix- metalloproteinase
MRI: magnetic resonance imaging
MUO: meningoencephalitis of unknown origin
NGAL: neutrophil gelatinase- associated lipocalin
rSpear: Spearman's rank correlation coefficient
SRMA: steroid- responsive meningitis- arteriitis
TNCC: total nucleated cell count
UTI: urinary tract infection.
